# Behaviours Associated with Acoustic Communication in Nile Tilapia *(Oreochromis niloticus)*

**DOI:** 10.1371/journal.pone.0061467

**Published:** 2013-04-19

**Authors:** Nicolas Longrie, Pascal Poncin, Mathieu Denoël, Vincent Gennotte, Johann Delcourt, Eric Parmentier

**Affiliations:** 1 Laboratory of Functional and Evolutionary Morphology, University of Liège, Liège, Belgium; 2 Laboratory of Fish and Amphibian Ethology, Behavioural Biology Unit, University of Liège, Liège, Belgium; 3 Aquaculture Research and Education Center (CEFRA), University of Liège, Tihange, Belgium; Fundação Oswaldo Cruz, Brazil

## Abstract

**Background:**

Sound production is widespread among fishes and accompanies many social interactions. The literature reports twenty-nine cichlid species known to produce sounds during aggressive and courtship displays, but the precise range in behavioural contexts is unclear. This study aims to describe the various *Oreochromis niloticus* behaviours that are associated with sound production in order to delimit the role of sound during different activities, including agonistic behaviours, pit activities, and reproduction and parental care by males and females of the species.

**Methodology/Principal Findings:**

Sounds mostly occur during the day. The sounds recorded during this study accompany previously known behaviours, and no particular behaviour is systematically associated with sound production. Males and females make sounds during territorial defence but not during courtship and mating. Sounds support visual behaviours but are not used alone. During agonistic interactions, a calling *Oreochromis niloticus* does not bite after producing sounds, and more sounds are produced in defence of territory than for dominating individuals. Females produce sounds to defend eggs but not larvae.

**Conclusion/Significance:**

Sounds are produced to reinforce visual behaviours. Moreover, comparisons with *O. mossambicus* indicate two sister species can differ in their use of sound, their acoustic characteristics, and the function of sound production. These findings support the role of sounds in differentiating species and promoting speciation. They also make clear that the association of sounds with specific life-cycle roles cannot be generalized to the entire taxa.

## Introduction

Many teleosts are able to emit sounds associated with different behaviours such as feeding competition [1], courtship [2], [3], [4] or agonistic behaviour [5], [6], [7]. More precisely, sound production can be used to deter intruders [8], [9], [10], to identify conspecifics [11], [12], [13], [14], or to attract and choose partners [15]. Moreover, sound characteristics can also inform conspecifics about the motivation [16], [17] or the size [18] of the caller.

In cichlids, sexual selection plays a central role in mate choice, meaning their complex courtship behaviours are considered to have a key role in their diversification process [19], [20]. In several species, courtship and agonistic interactions are closely associated with sound production [3], [21]. Moreover, these sounds are species-specific and allow species distinction based on acoustic characteristics such as trill duration, number of pulses per trill, pulse period, pulse duration, and interpulse interval [11], [12], [22], [23], [24]. There are today at least 29 species of Cichlidae described as sound producers, of which 25 are from Africa and 4 from the South American continent (Table 1). For some cichlid species, such as for *Archocentrus multispinosus*, several different types of sounds have been recorded and associated to specific contexts: burst and thump sounds are produced in agonistic situations and growls may help to synchronise partners during spawning. The less frequent “whoof sounds” have not yet been related to a specific behaviour [25].

**Table 1:** Summary of Cichlid species producing sounds referenced in the literature.

Species	Group	Type of sound	Sex	References
*Amantitlania nigrofasciata (1)*	NW	Br-r-r	♀	[27]
*Archocentrus centrachus (2)*	NW	Growls	♂–♀	[58]
		Thumps	♂	[10], [27]
*Archocentrus multispinosus (3)*	NW	Thumps	♂–♀	[25]
		Whoof	♂–♀	
		Growls	♂ (− ♀)	
		Volley sound	♂–♀	
*Astatotilapia burtoni (4)*	OW	Chewing	♂–♀	[59], [60]
*Haplochromis nyererei (5)*	OW	Quiver	♂	[15]
*Haplochromis omnicaeruleus (6)*	OW	Quiver	♂	[15]
*Hemichromis bimaculatus*	OW	Thumps	♂–♀	[27], [28]
		Br-r-r	♂–♀	
*Maylandia callainos (7)*	OW	Quiver	♂–♀	[11], [12], [20], [22], [61]
*Maylandia emmiltos (7)*	OW	Quiver	♂–♀	[11], [23]
		Moan	♂	
*Maylandia lombardoi (7)*	OW	Quiver	♂–♀	[52], [61]
*Maylandia zebra (7)*	OW	Quiver	♂–♀	[11], [12], [20], [22], [23], [43], [61], [62], [63]
		Quiver	♂–♀	
		Moan	♂	
*Maylandia « zebra gold » (7)*	OW	Quiver	♂–♀	[11], [12], [22]
*Mchenga conophoros (8)*	OW	–	♂	[13]
*Melanochromis auratus*	OW	–	♂	[20], [61]
*Melanochromis cyaneorhabdos*	OW	–	♂	[20], [61]
*Melanochromis johannii*	OW	–	♂	[20], [61]
*Oreochromis mossambicus (9)*	OW	Paired Burst Growls	♂	[2], [54], [64], [65], [66], [67], [68]
		Chewing	♂	
*Oreochromis niloticus (9)*	OW	Deep-pitched crack	♂–♀	[36], [37], [38]
*Pseudotropheus fainzilberi*	OW	Quiver	♂–♀	[11], [62]
		Moan	♂	
*Pterophyllum scalare*	NW	Tzz-tzz	–	[27]
*Pundamilia pundamilia*	OW	Quiver	♂	[15]
*Sarotherodon galileus*	OW	–	♂–♀	[25]
*Simochromis babaulti*	OW	Br-r-r	♂–♀	[69]
*Simochromis diagramma*	OW	Br-r-r	♂–♀	[70], [71]
		Chewing	♂–♀	
*Tilapia mariae*	OW	–	♂	[72]
*Tramitichromis intermedius*	OW	Quiver	♂	[13]
*Tropheus brichardi*	OW	Chewing	?	[71]
		Br-r-r	♂–♀	
*Tropheus duboisi*	OW	Chewing	?	[71]
		Br-r-r	♂–♀	
*Tropheus moorii*	OW	Chewing	?	[59], [71]
		Br-r-r	♂–♀	

NW = New world/OW = Old World.

*• = *valid name, *○* = Synonym(s).

*Cichlasoma negrofasciatum ○ = Amatitlania nigrofasciata •.*

*Cichlasoma centrarchus ○ = Archocentrus centrarchus •.*

*Herotilapia multispinosa ○ = Archocentrus multispinosus •.*

*Haplochromis burtoni ○ = Astatotilapia burtoni •.*

*Pundamilia nyererei ○ = Haplochromis nyererei •.*

*Neochromis omnicaeruleus ○ = Haplochromis omnicaeruleus •.*

*Pseudotropheus ○ = Metriaclima ○ = Maylandia callainos •, M. emmiltos •, M. lombardoi •, M. zebra •, M. « zebra gold » •.*

*Copadichromis conophorus ○ = Mchenga conophoros •.*

*Tilapia ○ = Sarotherodon ○ = Oreochromis mossambicus •, O. niloticus •.*

Although most occurrences of the production of sounds are reported among males, as for example in *Tramitichromis intermedius* [26] or *Maylandia spp* [11], females are also capable of emissions, as in *Archocentrus centrarchus* [10] or *Hemichromis bimaculatus* [27], [28].

Among the Cichlidae, the “Tilapia” group comprises species with oral incubation by females. In the breeding season, females brood the eggs until they hatch and the yolk sac is resorbed. The fry are then released and aggregate near their mother for *ca*. 21 days, re-entering her mouth in times of danger [29]. The agonistic behaviours [30], [31], [32], [33] and sexual behaviours [30], [34] of *Oreochromis niloticus* are well described: males defend their territories (arenas or leks) where they attract females. At the end of the mating sequence, the male quivers while circling the nest and is followed by the female, who takes both eggs and sperm into her mouth, where the eggs are fertilized [35].

The Nile Tilapia’s production of sounds has received little attention [36], [37], [38]. Males produce short-duration (250–400 ms), often double-pulse sounds. Most energy is below 200 Hz and includes three main low-frequency peaks [37]. The sound producing mechanism has been studied in adults [38]. It is made during a backward movement of the pelvic and pectoral girdles and a forward movement of the second pterygiophore of the anal fin. Muscle contractions should result in compression of the rib cage and the swim bladder.

Despite these studies, it has so far been unknown whether the female can emit sounds and whether there are links between specific visual behaviours and sound emission. The aim of this study was to test these hypotheses by an in-depth analysis of acoustic behaviour of *Oreochromis niloticus.* This work primarily relied on the known ethogram of *O. niloticus* males to determine which parts of this cichlid behaviour are related to sound production. In addition, we investigated different aspects of the behaviours of *O. niloticus* females and their ability to produce sounds.

## Methods

### Ethics Statement

Throughout the experiment, mortality was very low and did not exceed the average rate that we have observed in the species in captivity. The experiments were carried out under the approval of the Animal Care Committee of the University of Liège (forms 564 and 738) in accredited experimental rooms (LA 1610429 and LA 1610430).

### Fish

Nile tilapia *O. niloticus* (lake Manzala strain) used in the experiments were raised in the Behavioural Biology Unit (Laboratory of Fish and Amphibian Ethology), University of Liège (Liège, Belgium). They came from the Aquaculture Research and Education Center of the University of Liège (CEFRA-ULg, Tihange, Belgium). Adults (Total Length: 7 to 30 cm, sex ratio 1∶1) were stocked in a 4.5 m^3^ aquarium (375 cm×100 cm×125 cm) with coarse sand on the bottom where the males would dig their nests, removing the sand with their mouths [30]. Fish were fed *ad libitum* three times a week with commercial pellets (the excess was removed to avoid any pollution) and aquaria were maintained at 28°C with a photoperiod of 12L:12D (7 am –7 pm). The recordings of behaviours were performed in three 650L aquaria (200 cm×55 cm×60 cm) with the same environmental conditions. Fish stayed in these experimentation aquaria for 1 to 6 days, for the time of the recordings.

### Recording and Analysis of Sound Production

The first sound recordings were conducted in the stock aquarium, using a Digital Spectrogram Recorder (DSG, Loggerhead Instruments Inc, Sarasota, FL). A DSG is a low-power acoustic recorder designed to sample at different rates, continuously or on a duty cycle to a Secure Digital High Capacity (SDHC) memory. It makes possible the recording of scheduled periods at regular intervals. The rate of main sampling was 40 kHz, and the hydrophone (HTI) sensitivity was 186 dBV lPa^−1^. Prior to the recording sessions, all electric devices (ventilation, pumps and heating system) were unplugged.

Two recording series were conducted with the DSG:

- Nycthemeral variations: the number of sounds was recorded in the stock aquarium for 15 minutes every hour for four days, meaning 96 recordings were made for one group. The number of individuals in the stock tank was 94.
- Variations during “light” phase: another set of automatic recordings (15 minutes every hour during the light periods) was conducted for 8 days (N = 1, n = 8) in a 650L aquarium containing 15 males and 10 females.

Recordings were analysed with Avisoft-SASLab Pro version 4.38 (Berlin, Germany). Recording in small tanks induces potential hazards because of reflections and tank resonance [39]. A relevant equation [39] was thus used to calculate the resonant frequency of the stock and experimental tanks, and a low pass filter of 938 Hz (Stock) and 1.8 kHz (experimental) respectively was then applied to all sound recordings.

The sets of recordings were then automatically analysed with the function « Pulse Train Analyses » of Avisoft (Threshold = 0.1 V, Group Time 250 ms, Hysteresis 30 dB). A manual count was performed for several portions of the sound ranges to verify the validity of the automatic analysis.

### Recording of the Video Sequences

In order to observe a possible association between the sounds produced and visual behaviour, sounds were recorded with a hydrophone HTI (sensitivity 186 dB re. 1 V µPa^−1^) connected to a digital video camera Canon FS100. Prior to the recording sessions, all electric devices (ventilation, pumps and heating system) were unplugged, and the hydrophone was placed vertical to the nest of a territorial male at least 20 min before recording in order to minimise any impact on behaviour.

#### 1. Males vs. Males - Agonistic interactions

Oreochromis niloticus territorial males were observed for 6 days in two different types of territorial situations. The aim was to learn in what ways the presence of other males induced sound production by focal territorial males.

- First observations were made to study the behaviour of a territorial male towards non-territorial males and to seek information about the influence of the population density on the emission of sounds by the territorial males. This experiment was carried out with 7 different territorial males. A male (SL mean ± SD, 28.6±6 cm, n = 7) was first placed in an aquarium two days before starting the recordings. This period was necessary to give it time to delimit its territory. Intruders (SL mean ± SD, 24.4±6 cm, n = 28) were then added one after the other in the tank at the rate of one fish per day. A total of 4 intruders were introduced in each of the sessions. Each day, the sounds and the behaviours were recorded by sessions of 15 minutes that started 5 minutes after the introduction of the intruder. A total of 28 (7 males×4 intruders) recording sessions were carried out.
- The second set of observations examined confrontations between territorial males. First five males were simultaneously introduced into the experimental tank. Three of them (SL mean ± SD,11.7±0.6 cm) took possession of a zone and then dug and defended an arena. The two remaining males were then removed from the tank. The three territorial males were acclimated for two days before starting the recording sessions. During recording sessions, the hydrophone (connected to the video camera) was placed over each male’s arena successively. Each recording session occurred between 11 am and 3 pm and lasted 15 minutes. The elapsed time between two adjacent sessions was at least one hour. A total of 4 sessions per male were recorded and analysed.

#### 2. Males vs. Females – Courtship interactions

First one male (n = 4, SL mean ± SD,18±8 cm) was placed in the experimental tank for two days before recording in order to allow him to acquire the status of territorial male. Three females (SL mean ± SD, 13.7±7 cm, n = 12) were then added to provoke courtship behaviour. Females (n = 12) were chosen according to the swelling of their genital papilla. Experiments were conducted four times. In each experiment, sounds and behaviours were recorded as long as the courtship behaviour and the mating lasted (from 20 to 60 min). At the end of the experiment, 12 complete reproduction cycles were recorded.

#### 3. Females vs. Females - Territoriality and Oral incubation

Experiments with “female *vs.* female” interactions were carried out to determine if females are capable of sound production. Five females (SL mean ± SD, 12.1±1 cm) were placed in each of three experimental aquaria. Sounds and associated behaviours were then recorded during 3 sessions lasting 10 minutes each. Female behaviours associated with sound production were listed.

#### 4. Females – Parental care

This set of observations was devoted to looking for sound production during females’ oral incubation. For this experiment, a female incubating eggs or fry was exposed to intruders such as *Amphilophus citrinellus* (South American cichlid) and to females of the same species. Five different females were tested, allowing 55 experimental encounters (n = 55) that each lasted 10 minutes. Twenty-three encounters were arranged between *O.niloticus* females and 32 between female *O. niloticus* and female *Amphilophus citrinellus.*

### Analysis of the Video Files

The various behaviours analysed are listed in Table 2. The association between sounds and broadcasting fish can be made visually. Sounds are made during a backward movement of the pelvic and pectoral girdles [38], but it was not possible to observe the calling fish in every experimental test.

**Table 2:** Description of *Oreochromis niloticus* behaviours according to [2], [30], [34], [35], [57], [73].

Behaviours	Brief description		
**Agonistic behaviours: Threat**			
Lateral Display	The fish shows the opponent his flank. The dorsal fin is raised. When threat becomes intense, the animal can bulge the branchiostegal membrane and spread his opercles.		
Frontal Display	The two opponents approach each other frontally with or without bulging the branchiostegal membrane and spreading their opercles.		
Tail Beating	In a parallel or anti-parallel position, opponents undulate the entire body. The branchiostegal membrane is bulged and opercles are spread.		
Chase	Rapid swimming towards a threat.		
Charge	Rapid parallel swimming of the two opponents towards each other.		
**Agonistic behaviours : Attack**			
Lateral Attack/Butting	Lateral display with contact of the mouth (open or closed) of one opponent against the other.		
Frontal Attack	Frontal display where the assailant tries to bite the other opponent, which responds by opening his mouth as well.		
Mouth fighting	Frontal attack where the two opponents press their mouths together, followed by a pulling/pushing game between the two fishes.		
**Pit Related Activities**			
Nest digging		Digging of the nest by the territorial male.	
Nest hover		Male hovers motionless in his nest.	
Nest display		Spreading of the dorsal and pectoral fins	
**Courtship behaviours**			
Tilting		The male comes in front of the female, this head angled towards the floor. All the fins, with the exception of the pelvic fins, are fully spread.	
Leading		In a tilting position, the male directs the female to the centre of his territory.	
Lateral display		The male spreads all his fins. The branchiostegal membrane is strongly tensed by the spreading of the operculum and the lowering of the hyoid arch.	
Tail wagging		In a lateral display, the male “strikes” his caudal towards the female.	
Nuptial dance		The male zigzags and undulates his body in front of the female, with the caudal, anal and dorsal fins partially spread. The branchiostegal membrane is completely slack.	
Digging		Similar to the digging behaviour during the establishment of the nest. Courted female can take part in this behaviour.	
**Parental care: Mouthbrooding**			
Charge		Rapid parallel swimming of the two opponents towards each other.	
Chase		Rapid swimming towards a threat.	
Lateral Attack/Butting		Lateral display with contact of the mouth (open or closed) of one opponent against the other.	

Video films were analysed with Noldus Observer Video Pro 4.1 software [40], [41]. This software made possible the analysis and recording of events (instantaneous behaviour such as sound production or bites) or states (behaviours with appreciable duration such as arena digging or frontal display) by typing keys from the keyboard in synchronization with video-files. The different behavioural patterns were consistent with previous descriptions of *O. niloticus* (Table 2).

### Analysis of the Sound Productions of Females

Sounds were digitized at 44.1 kHz (16-bit resolution) and analysed with the Avisoft-SASLAB Pro 4.33 software [1024-point Hanning window fast Fourier transform (FFT)]. Only sounds with a high signal-to-noise ratio were used in the analysis. Temporal features were measured by oscillograms and frequency variables from power spectra (filter bandwidth 300 Hz, FFT size 256 points, time overlap 96.87% overlap and a flat top window). The sound parameters measured were: sound duration (ms); number of pulses in a sound; pulse period (measured as the average peak-to-peak interval between consecutive pulses in the entire sound, ms); pulse length (measured as the time from the beginning of one pulse to its end, ms); and dominant (or main) frequency, representing the most intense frequency (in Hz).

### Statistical Analysis

The statistical analysis was made with Statistica 9.1. The Shapiro-Wilk W-test was used to test the normality of the data. A Wilcoxon’s non-parametric paired test was used to determine if the fishes’ visual behaviours were significantly more often accompanied by sounds or lack of sounds. We used this paired test because the data of behaviours with and without sounds are dependent. A Spearman rank correlation coefficient (R_S_) was used to test the relation between the number of intruders and the number of sound produced. The chi-square test was used to determine if sounds were more frequently associated with some behavioural patterns than others. The non-parametric Kruskal-Wallis one-way ANOVA by ranks with subsequent Dunn's test for pairwise comparisons was used to compare the rate of bites and production of sound in three contexts (territorial males ♂♂ *vs.* intruders ♂; ♂♂ *vs.* ♂♂; ♂♂ *vs.* ♀). The Mann-Whitney U-test was used to compare the sounds/behaviour co-occurrence between the various contexts; and the Student t-test was used to compare the duration of the sound, the number of pulses, the pulse duration and the main frequency of sounds in the experimental conditions.

## Results

### Nycthemeral Rhythm of the Production of Sounds

**Figure 1:**
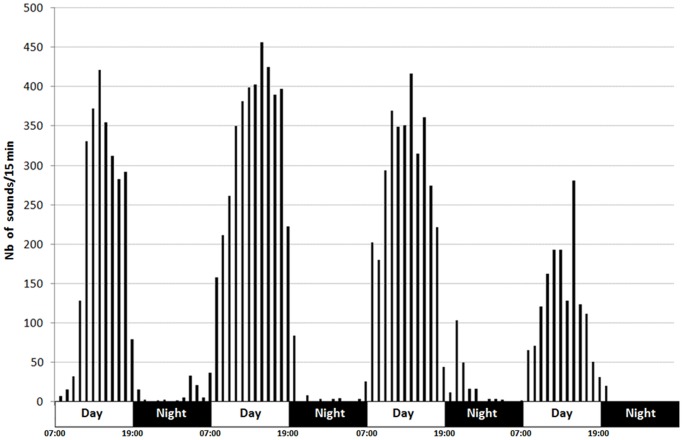
Nycthemeral production of sounds in *Oreochromis niloticus*. Number of fishes = 94.

**Figure 2:**
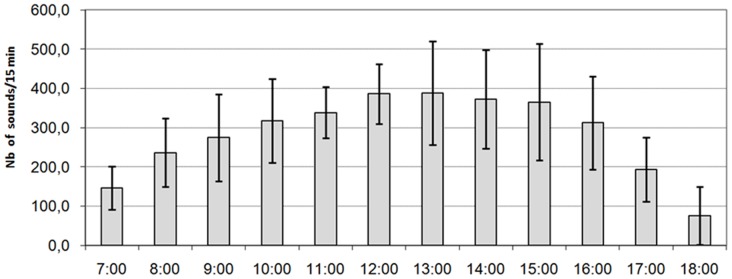
Diurnal production of sounds in *Oreochromis niloticus*, mean ± SD (n = 8 days, N = 25 fishes including 15♂ and 10♀).

The sound production cycle of male *Oreochromis niloticus* coincided with the nycthemeral cycle: the sound production took place mainly during the diurnal phase (Fig. 1), but some sounds were also recorded during the night. However, the rate of sound production is not uniform at all stages of the light phase (Fig. 2). Sound production regularly increased throughout the first 7 hours of illumination (until, essentially, the beginning of the artificial afternoon) and then decreased until the illumination ceased (nightfall).

### Association of Behaviours with the Production of Sounds

#### a) Acoustic behaviours between males

The confrontation between territorial males and non-territorial intruders resulted in a range of behaviours that were sometimes associated with sounds (Table 3). Other behaviours (see Table 2) also resulted which were not accompanied by sound production.

**Table 3:** Mean ± SE of the occurrence of a behaviour appearing on a 15 min recording, for *Oreochromis niloticus* territorial males.

Behaviours	♂♂ vs ♂ (1 Territorial Male x Intruders; N = 7, n = 28)			♂♂ vs ♂♂ (3 Territorial Males; N = 1, n = 12)		
	Silent	Sound	Wilcox	Silent	Sound	Wilcox
Frontal Display	–	–	–	2.42±0.57	4.58±1.05	ns
Mouth Fighting	1.4±0.4	–	–	3±0.8	0.5±0.2	**
Lateral Display	13.9±1.3	5.51±1.4	***	0.8±0.2	0.2±0.2	ns
Nest Hover	10.4±0.9	0.3±0.17	***	5.3±1.3	7.6±1.2	ns
Nest Digging	4.8±1.1	0.4±0.3	***	2.6±1	2.4±1	ns
Nest Display	18.2±1.6	3.9±1.1	***	1.5±0.2	1.3±0.4	ns
Chase	8.58±1.53	0.21±0.12	***	16.5±2.78	13.3±3.6	ns

The observed behaviour was silent (Silent) or accompanied by sonic emissions (sound). Results of the Wilcoxon test:

* p<0.05,

*** p<0.001.

♂♂ = territorial males;

♂ = intruder males.

**Figure 3:**
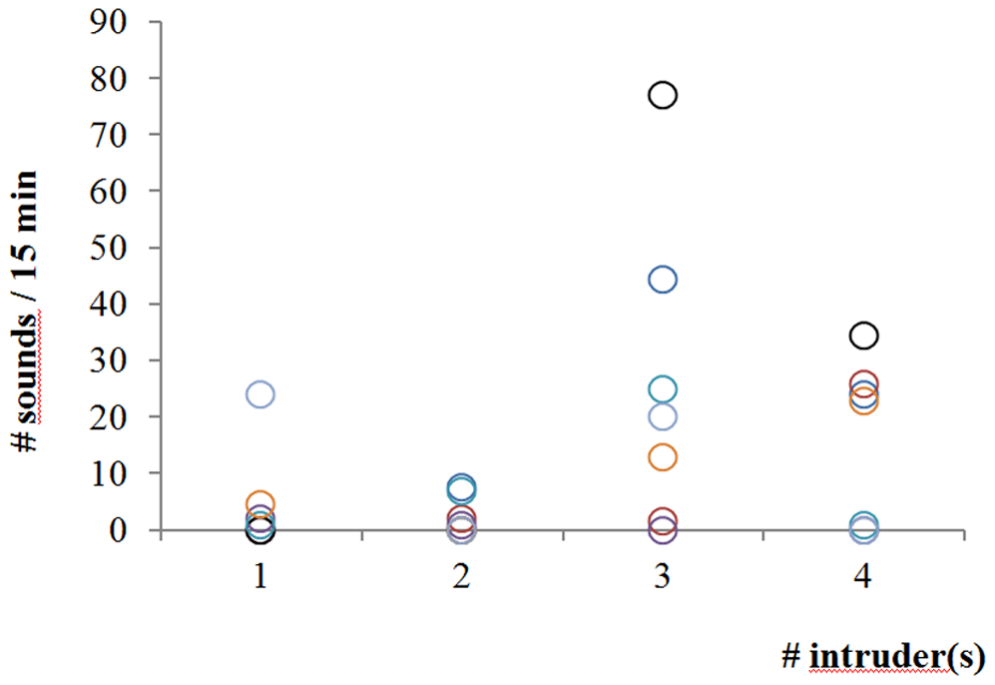
*Oreochromis niloticus* - Number of sounds produced during 28 recording sessions of 15 minutes with 1 to 4 male intruders in the tank.

There was no relation between the number of sounds and the number of intruders present in the tank (Spearman correlation, R_S = _0.34; p>0.05) (Fig. 3). In confrontations between a territorial male and intruders, the different behaviours were usually performed without sound production (Table 2), indicating that sounds do not constitute the essential part of these various behaviours. Comparisons were realised at two levels. 1) First we compared co-occurrence of sounds and behaviours between the different kinds of behaviour within each kind of meeting to know which behavior is more commonly associated with sounds. During the first set of experiments (Territorial Male ♂♂ *vs* intruders males ♂), some behavioural patterns (mainly Lateral display and Nest display) were more associated with sounds than others (see Table 4).

**Table 4:** Proportions of behavioural patterns exhibited in association with sounds in two contexts: **♂♂ vs. ♂** (1 Territorial Male x Intruders; above the diagonal) and **♂♂ vs ♂♂** (3 Territorial Males x Intruders; under the diagonal).

	♂♂ vs ♂	♂♂ vs ♂♂	Frontal display	Mouth fighting	Lateral Display	NestHover	NestDigging	NestDisplay	Chase
Frontal display	–	65.5%		–	–	–	–	–	–
Mouth Fighting	–	14.3%	***		–	–	–	–	–
Lateral Display	28.3%	–	–	–		***	***	***	***
Nest Hover	2.8%	58.7%	ns	***	–		*	***	ns
Nest Digging	8%	48.9%	ns	***	–	ns		*	*
Nest Display	17.8%	50%	ns	**	–	ns	ns		***
Chase	2.4%	44.9%	***	***	–	**	ns	ns	

ns: p>0.05,

* p<0.05,

** p<0.01,

*** p<0. 0.001 (chi-square tests).

Statistics are shown only for occurrences for behaviours higher than 10.

In the second set of experiments (Territorial Male ♂♂ vs Territorial Male ♂♂), the confrontation between a focal individual and other territorial males elicited an additional threatening behaviour: the frontal display during which two opponents approach each other frontally while swelling the branchiostegal membrane and spreading their opercles. Although mouth fighting is more often associated with sounds (14.3%) in the second set of experiment, the co-occurrence remains significantly less important than in other behaviours (Table 4).

**Figure 4:**
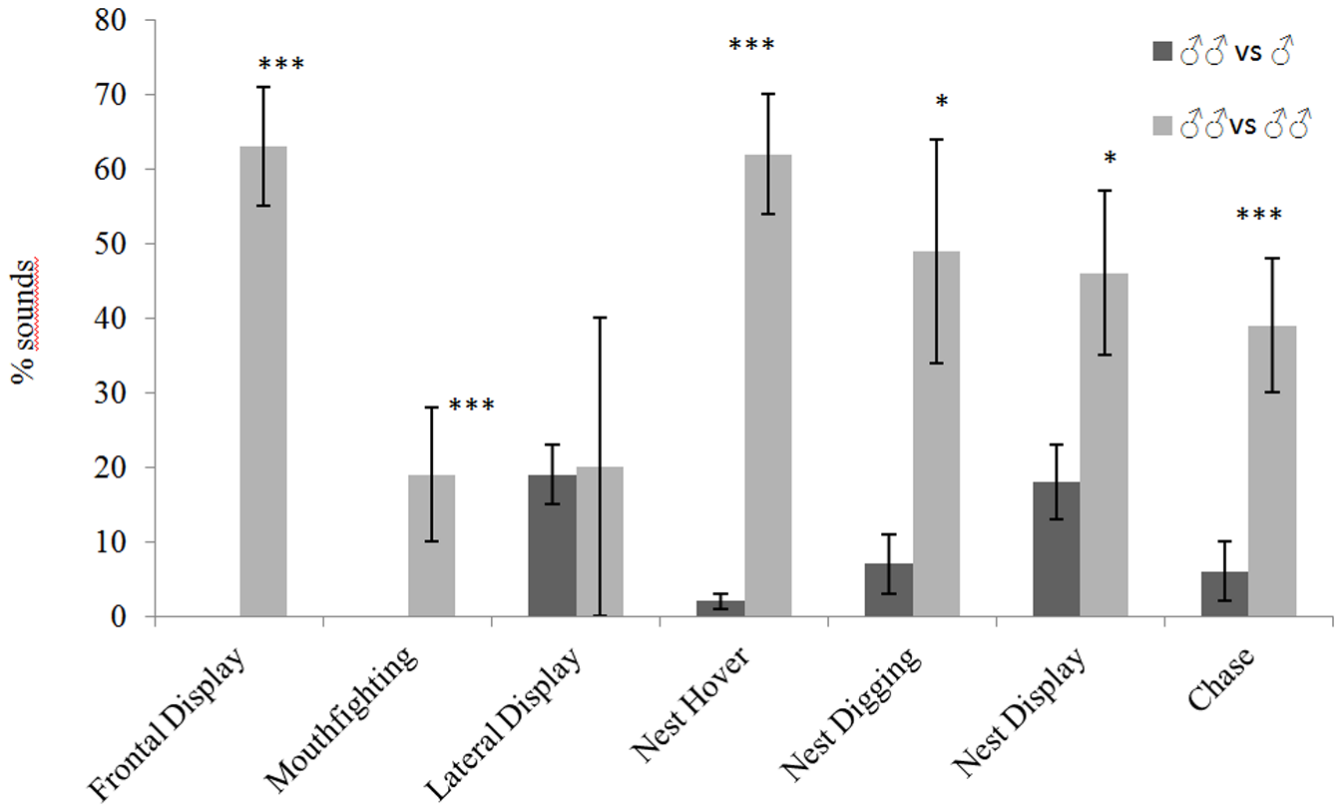
Histogram of the average percentage (mean ± SE) of sound production during the display of different behaviours in *Oreochromis niloticus* males during different kinds of encounters. * p<0.05, *** p<0.001: significant differences between light and dark grey bars (Mann-Whithney test). ♂♂ = territorial male; ♂ = intruder male. The statistical test cannot cannot be applied to Frontal display and Mouth fighting because these behaviours were not recorded in ♂♂ vs ♂.

Faced with non-territorial intruders, the territorial males mostly produced their threats from the nest. Conversely, confrontations with other territorial males mostly resulted in chases and frontal displays, and lateral displays became rare (Table 3). All these behaviours seem to correspond to a more aggressive response to other territorial males. However territorial males bite non-territorial intruders more often than other territorial males (Table 5). In comparison with encountering between territorial male and non-territorial intruder, the sound production of a territorial male encountering other territorial males was associated with a greater number of different behaviours, and the sound production rate was six times greater (Table 5). 2) In the second set of comparisons, we compared the co-occurence of behaviour and sounds between males placed in two different kinds of meeting (Table 6). In the presence of other territorial males, sounds were emitted simultaneously with 19%–63% of the occurrences of the different behaviours (Table 6, Fig. 4). More precisely, sounds were more associated with Frontal display, Nest hover and Nest display (Table 6). This co-occurrence between sound production and behaviour display was less than 20% when the territorial male was confronted with non-territorial males (Table 6, Fig. 4). During meetings between territorial males, more sounds were significantly realised except during Lateral display (Table 6).

**Table 5:** Mean rate (± SE) per minute of event-behaviour during confrontation of territorial males against intruders (♂♂ vs. ♂, N = 7, n = 28), between territorial males (♂♂ vs. ♂♂, N = 3, n = 12) and territorial males against females (♂♂ vs. ♀, N = 6, n = 24).

Behaviours	♂♂ vs ♂	♂♂vs ♂♂	♂♂vs ♀
**Sounds**	0.9±0.2	6±1.7	0.5±0.2
**Bitings**	1.7±0.2	0.1±0.02	0.6±0.15

**Table 6:** Mean percentage (± SE) of co-occurrence between behaviour and production of sounds.

Behaviours	♂♂ vs ♂ N = 7	♂♂ vs ♂♂ N = 3	Mann - Whitney
Frontal Display	–	63±8	–
Mouth Fighting	–	19±9	–
Lateral Display	19±4	20±20	ns
Nest Hover	2±1	62±8	***
Nest Digging	7±4	49±15	*
Nest Display	18±5	46±11	*
Chase	6±4	39±9	***

Results from the Mann-Whitney test. ns: p>0.05,

* p<0.05,

*** p<0.001.

♂♂ = territorial male;

♂ = intruder male.

#### b) Acoustic behaviour between males and females

Observation of the confrontations staged between a territorial male and one or several females showed there were no sounds associated with reproductive behaviours (such as tilting, leading, lateral display, tail wagging and the wedding dance, see Table 2) and spawning. However, males emitted sounds towards females in the context of territorial defence.

During the three kinds of meetings (Table 5), call rates appeared to be significantly different (Kruskal-Wallis**,** H = 19.4; N = 79, p>0.05). However, pair-wise comparisons indicated the global rate ( = 0.5 sound/min) of sound production of territorial males towards females corresponded to the rate of sound production of territorial males ( = 0.9 sound/min) when facing non-territorial male intruders (Dunn’s test, p>0.05). These rates were both significantly lower (Dunn’s test, p<0.05) than the rate of 6 sounds per minute when facing other territorial males (Table 5). The difference in biting rates towards the two groups was also tested (Kruskal-Wallis Test, H = 27.3; N = 79). Territorial males bite females (0.6 bites/min) significantly less (Dunn’s test, p<0.05) than non-territorial intruders (1.7 bites/min). However, the biting rate toward females and other territorial males was similar (Dunn’s test, p>0.05).

#### c) Acoustic behaviour of females

Females were recorded in three situations: Territorial (the female digs and defends an arena); Incubation (eggs in the mouth); and Protection (free alevins that can be taken back in the mouth in case of danger). In *Oreochromis niloticus*, females emitted sounds in two contexts: Territorial and Incubation.

**Figure 5:**
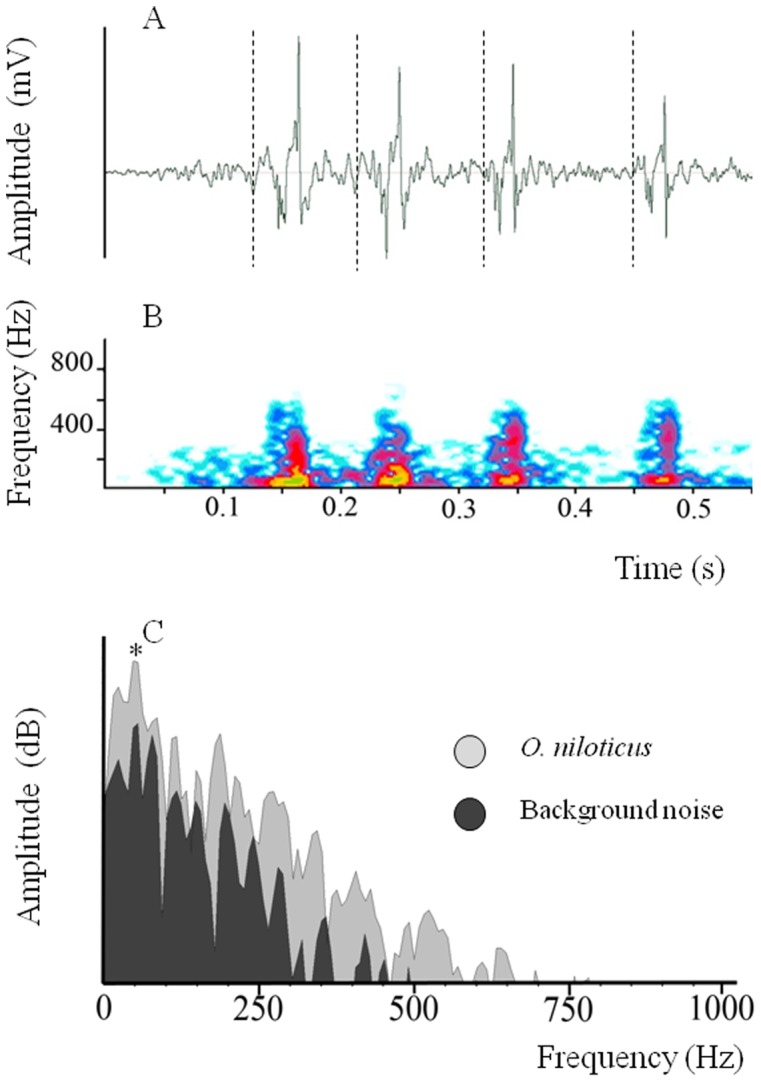
Oscillogram (A) and spectrogram (B) of a sound having four pulses in *Oreochromis niloticus* female. Frequency spectrum of a pulse (C). (*) = Main frequency. Spaces between dotted lines correspond to pulse period.

The 315 sounds (recorded from 7 different territorial females) lasted for 320 ms ±101 (Χ ± SD) and consisted of 3.1±1 pulses (Table 7, Fig. 5). Pulse periods had an average length of 105±23 ms, and the sounds had a frequency of 49±14 Hz (N = 7, n = 963). For females in incubation, the sound duration was 264±84 ms with 2.5±0.6 pulses (N = 4, n = 141, Table 7). Pulse periods had an average length of 107±21 ms, and the sounds had a frequency of 48±15 Hz (N = 4, n = 348). The comparison between the data obtained in the two behavioural contexts showed the sounds were significantly different in their duration and their number of pulses. However, pulse length was not significantly different between the two contexts; the difference between sounds was only due to the number of pulses (Table 7).

**Table 7:** Means (± SE) of the sonic parameters of *Oreochromis niloticus* females, recorded in situation of territoriality (Nest) and of oral incubation (Eggs).

	n	Nest (N = 7 females)	n	Eggs (N = 4 females)	t-Test (p<0.05)
**Sound length (ms)**	314	320.7±101	141	264.8±84	***
**Nb of pulses/sound**	314	3.1±1	141	2.5±0.6	***
**Pulse duration (ms)**	963	105.1±23	348	107.3±21	ns
**Frequency (HZ)**	963	49.6±14	348	48.3±16	ns

*** p<0.001.

**Figure 6:**
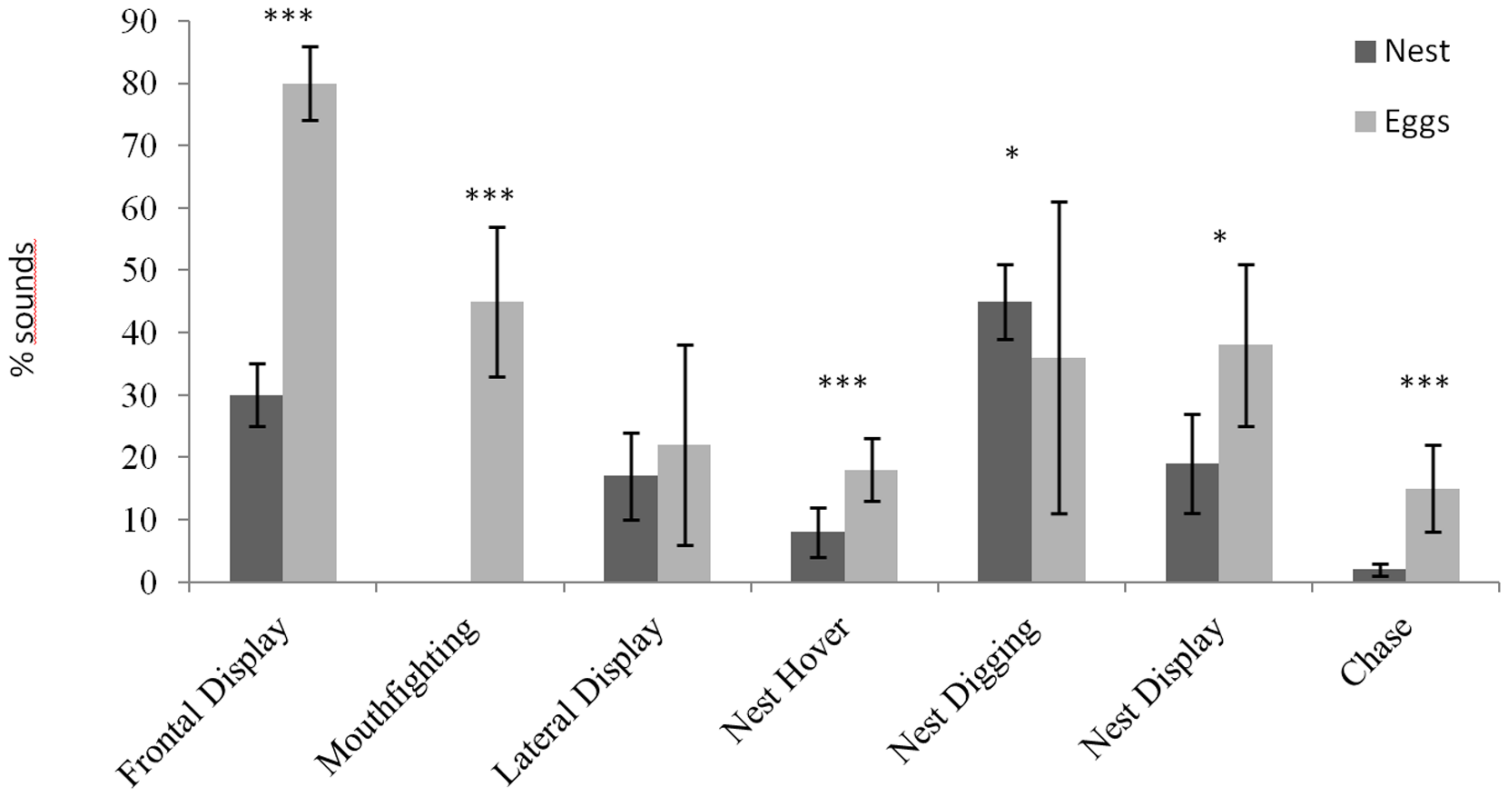
Mean percentage (% ± SE) of sound production during the display of different behaviours in *Oreochromis niloticus* females. Nest = territorial female; eggs = female incubating eggs. * p<0.05, *** p<0.001: significant differences between light and dark grey bars (Mann-Whithney test). The statistical test cannot be applied to Mouth fighting because this behaviour was not recorded during “Nest” experiments.

The female acoustic behaviour associated with territory defence was very similar to that of the males. Females were also able to produce sounds during behaviours associated with territorial defence, except during mouth fighting (Table 8). Moreover, as with the males, sounds were not made systematically during the different behaviours (Table 8). They mostly occurred during nest digging and frontal display, with sounds accompanying 45% and 30% of the behaviours respectively, whereas fewer than 20% of other behaviours were associated with sounds (Table 9, Fig. 6).

**Table 8:** Mean (±SE) of the occurrence of a behaviour appearing on a 15 min recording, for *Oreochromis niloticus* females.

Behaviours	Nest (N = 7 females, n = 26)			Eggs (N = 4 females, n = 19)		
	Silent	Sound	Wilcoxon	Silent	Sound	Wilcoxon
**Frontal Display**	8.1±1.3	4.2±0.9	*	1.1±0.3	3.7±0.7	**
**Mouth Fighting**	1.5±0.5	–	–	1.6±0.5	1.4±0.4	ns
**Lateral Display**	2.1±0.4	0.4±0.2	**	1.5±0.4	0.7±0.5	ns
**Nest Hover**	7.6±0.9	0.6±0.3	***	8±1.6	1.3±0.4	**
**Nest Digging**	5.7±0.7	5.4±0.9	ns	7.3±3.3	3±1.5	ns
**Nest Display**	2.6±0.6	0.5±0.2	**	8.4±2.4	0.9±0.2	*
**Chase**	4.7±1.2	0.2±0.2	**	3.5±0.9	0.5±0.2	**

Behaviours were silent or accompanied by sounds. Wilcoxon test: NS p>0.05,

* p<0.05,

** p<0.01,

*** p<0.001.

Nest = Territorial female; Eggs = Female incubating eggs.

**Table 9:** Mean percentage (± SE) of co-occurrence between a behaviour and a production of sounds.

Behaviours	Nest (N = 7 females, n = 26)	Eggs (N = 4 females, n = 19)	Mann - Whitney
Frontal Display	30±5	80±6	***
Mouth Fighting	–	45±12	***
Lateral Display	17±7	22±16	ns
Nest Hover	8±4	18±5	***
Nest Digging	45±6	36±25	*
Nest Display	19±8	38±13	*
Chase	2±1	15±7	***

Mann-Whitney test: NS p>0.05,

* p<0.05,

*** p<0.001.

Nest = territorial female; Eggs = Female incubating eggs.

Females were also able to make sounds during the oral incubation of their eggs (Table 10). In this case, sound productions co-occurred more often during Frontal Display than during the other behaviours (77%, Table 10). In addition, sounds were associated with 45% of Mouth Fighting by incubating females, but territorial females did not use this behaviour (Tables 9 and 10).

**Table 10:** Proportions of behavioural patterns exhibited in association with sounds in two contexts: Nest = Territorial female (above the diagonal); Eggs = Female incubating eggs (under the diagonal).

	Nest	Eggs	Frontal display	Mouth fighting	Lateral Display	NestHover	NestDigging	NestDisplay	Chase
Frontal display	33.9%	77.2%		***	***	***	ns	***	***
Mouth Fighting	0%	45%	**		ns	ns	**	ns	ns
Lateral Display	17%	26.7%	***	ns		*	***	ns	*
Nest Hover	7.6%	14.3%	***	***	ns		***	*	ns
Nest Digging	48.6%	26%	***	ns	ns	*		***	***
Nest Display	17%	9.8%	***	***	ns	ns	**		*
Chase	5.1%	14.6%	***	**	ns	ns	ns	ns	

ns: p>0.05,

* p<0.05,

** p<0.01,

*** p<0. 0.001 (chi-square tests).

Comparisons between territorial and incubating females showed other differences in behaviours as well. Although the same behaviours were realised during nest defence and egg defence, more sounds co-occurred with behaviours when the female was incubating (Table 8). The digging behaviour (Digging Nest) reported for incubating females was actually observed shortly after spawning and then disappeared rapidly.

#### d) Acoustic behaviour during female parental care

During fry guard, no sound was heard either to confront the intruder (*Amphilophus citrinellus* or *Oreochromis niloticus*) or to call fry to shelter inside the oral cavity. Faced with an intruder, females chose to attack rather than to discourage it by threats, quickly eliminating the danger. After the attack, the female retrieved her fry into her mouth.

## Discussion

Producing signals represents an economic way to solve disputes over resources. According to Ladich and Myrberg [6], defence and offence begin with signals, before physical interactions occur. In *O. niloticus*, meetings always include visual displays, but acoustic displays are only produced during escalated contests. Acoustic display appears to be only a part of a complex signalling system during interactions.

### Rate of Sound Production

Sound productions in *Oreochromis niloticus* take place mostly during the day (Fig. 1). All the sounds recorded during this study accompanied previously known behaviours, and no particular behaviour was systematically associated with sound production. This means that sounds are mostly produced to emphasize or reinforce visual behaviours that are firstly mainly based on visual stimuli [42], [43].

In *O. niloticus*, reproduction usually occurs in the afternoon and evening [31], while the early hours of the day are assigned to nest making/improvement and to defence of territory against other males attempting to settle down. Falter [31] showed that the territorial behaviour of *O. niloticus* was subject to variation during the day. In a study of 200 males in an aquarium (4.85 m^3^), the number of defended arenas increased during the day from about 56 between 8 and 11 am to 123 between 2 and 5 pm [31]. These data are in complete accord with the rates of sound production observed in the present study, which also increased gradually, with a peak in emissions at around 1 pm (or 7 hours post-lighting). Early in the day, the establishment and defence of a first arena do not face hostility from congeners. However, those males subsequently establishing arenas meet aggression from the territorial males already installed, increasing the global sound rate of the community. Territorial behaviours are accompanied by sounds, and the rate of sound production is more frequent in an aquarium where several territorial males are present. The increase in sound production during the day could therefore result from the increasing number of individuals who acquire or seek to acquire a territorial status as spawning time approaches.

The difference between vocalizing activity during the day and at night could possibly be simply a side-effect of the lack of visual stimuli during the night. In cichlids visual cues are important in mate choice, in male-male interactions and in non sexually motivated communication such as parent-offspring communication or species recognition [44], [45]. This would reinforce the hypothesis that acoustic displays are mainly used to support visual cues; sounds seem to carry messages only when they are associated with visual displays.

As in *Amphiprion* species, no sound production is involved in reproductive behaviour [46]. We have tested 4 different males in a total of 12 reproduction cycles. The number of tested males could seem low but in other cichlid species such as *O. mossambicus* [2], all the territorial males made sound during reproduction, and not only some of them. Moreover, males made sounds during other behaviours meaning they were able to produce sounds. Finding in the future some *O. niloticus* males are able to produce sounds during reproductive behaviour could in fact reinforce the main results of this study: sounds are produced to reinforce visual behaviours in *O. niloticus*. However, the number of sounds in the altercations between males might be a criterion for mate choice. Indeed, the simplest message that can be conveyed by the sounds is the location of a breeding area and the motivation of its owner [47]. In the cichlids *Haplochromis nyererei*, *H. omnicaeruleus* and *Pundamilia pundamilia*, females significantly prefer males who are associated with sound production [15].

### Sound Production by Territorial Males

In the cichlid *Archocentrus centrarchus*, acoustic communication was considered an inhibitor of aggressiveness because the sounds of territorial males reduced the number of physical injuries during aggressive encounters related to territory defence and progeny protection [48]. In *Maylandia zebra* (Cichlidae), aggressive behaviour is based on visual stimuli, and playback experiments have showed that acoustic signals alone never trigger aggression. Furthermore, when fish can only interact visually the association between visual and acoustic channels seems to lower the level of aggression observed [43]. However, as summarised by Ladich and Myrberg [6], sounds can also repel an intruder, increase an opponent’s aggressiveness or be used to assess a competitor’s fighting ability.

Sound production in *Oreochromis niloticus* territorial males is associated with behaviours of threat (frontal display, nest display, etc.) and chase (Table 2). When the territorial male is opposing dominated intruders, simple assault or threatening movements seems to be enough to keep them away from the arena. However, faced with other dominant males, more sounds are associated with threatening behaviours (Tables 3, 4 and 5). In *O. niloticus*, there are simultaneously more sounds and fewer bites between territorial males having a high status than between territorial males and females or non-territorial males. Once again, the major role of the sounds in *Oreochromis niloticus* seems to be to reinforce the messages of the visual stimuli.

In the sister species *O. mossambicus* [49], [50], sounds are emitted in all phases of courtship, but especially during late stages of courtship including spawning. In the late stage of courtship, the display called “Tail Wagging” (tail-flick) is associated with sounds in 89.7% of the behaviours. Amorim *et al.* [2] suggested that the acoustic emissions in this species may play a role in signalling the presence and reproductive readiness of males and in synchronizing gamete release, as in the cichlid *Tramitichromis intermedius* [26]. No sound production is associated with aggressive behaviours in *O. mossambicus* [2]. In *O. niloticus*, sounds are principally associated with territory defence and threatening purposes but not with courtship/mating behaviours *per se*. Nevertheless, because emissions start during nest building, it may be the case that females use sounds to gain information on the reproductive status of the males. *Oerochromis niloticus* also has a lower number of pulses (2–5 *vs.* at least 10), a longer pulse duration (150 *vs.* 10 ms) and a lower peak frequency than *O. mossambicus* [2], [37], allowing the two acoustic signatures to be easily differentiated. Yet while the pulse duration between the two species is quite different, the strong frequency component peaking at *ca.* 40 Hz in both is otherwise not common in fish sounds, which suggests they have similar sound producing mechanisms [38].

Although in an artificial environment hybridizations are always possible between these two sister-species, they are rare (or poorly documented) in the wild [47], [51]. According to a study by N’Gokaka [34], mating between *O. niloticus* and *O. mossambicus* can only occur shortly after individuals meet because there is confusion of sexual calls. Indeed, the divergences in their reproductive patterns make reproduction even less likely as the heterospecific partners may recognise each other easily. These matings are advanced by a high state of sexual motivation in females and only a brief encounter before spawning, two conditions making the partners more likely to respond to inappropriate stimuli. The presence (*O. mossambicus*) or the absence (*O. niloticus*) of mating sounds increases the differences in their behavioural patterns. The difference between these two species might be a factor that has contributed to their specific isolation by reducing errors in reproduction between heterospecific partners.

### Production of Sounds Among Females

This is the first study to highlight the ability of *O. niloticus* females to produce sounds. However, this ability has already been noted in other Cichlidae species (Table 1) where females produce sounds in aggressive behaviours and protection of spawn. The characteristics of the female calls are similar to those observed in males [37]: sounds are composed of few pulses (generally <5), for which the period is *ca.*100 ms and the main frequency between 30 and 100 Hz (Fig. 5). Because sexual dimorphism of morphological characteristics is extremely low in this species, both sexes should use the same sound-producing mechanism. Moreover, the ability of the incubating females to make sounds reinforces the hypothesis that pharyngeal jaws are not involved in sound production in this species [38] because having eggs in the mouth might preclude sound production by this mechanism.

In *O. niloticus* territorial females sound productions are associated with similar behaviours to those reported in territorial males (♂♂ vs. ♂♂) but are less frequent, with the exception of Nest digging. The digging of an arena by a female is usually observed before spawning in aid of the territorial male [30], but females may also dig an arena alone [42], [52], [53]. The female that digs an arena (while emitting sounds) usually spawns within 2–3 days and then incubates the eggs. During this incubation, the female may continue to issue sounds, probably to protect her eggs. Similar observations have been made of females of other Cichlidae species, such as *Archocentrus centrarchus* and *Hemichromis bimaculatus*, that emit sounds to protect their eggs, but also while preparing the nest for spawning [10], [28]. In *Maylandia zebra*, Simões *et al*. [23] showed that the sounds were produced by females when they were sexually receptive (visible ovipositor) or in oral incubation. Similar observations of sound productions in females in the state of oral incubation have been documented in *Oreochromis mossambicus* [54].

Comparison of the rate of sounds/behaviour co-occurrence between a territorial female and an incubating one indicates a significantly higher number of sound productions during frontal threats and mouth fighting in the incubating female. This difference may reflect the changing role of the mouth between a territorial female and an incubating one. For the territorial female, the mouth is available to take part in defence and combat. The incubating female uses her mouth to protect her eggs. When she is doing so, mouth fighting behaviour is hazardous because she risks losing the spawn. Sound emissions may therefore be used in order to curtail a potentially dangerous situation for the eggs. In this context, the production of sounds may be being used to inhibit the aggressiveness of the opponent. This hypothesis is reinforced by studies of *Amphilophus centrarchus,* a substrate incubator, which report that the females emit more sounds against threats after spawning (during incubation) [10].

### Parental Guard by Females

In the animal kingdom the degree of aggressiveness of females increases during the phase of parenting for animals as diverse as rodents [55] and crustaceans [56]. This increasing aggressiveness in females helps in protecting their offspring from predators [57]. In teleosts, the quickness and intensity of aggressions are influenced by both size and age of the progeny. In the Mozambique tilapia *Oreochromis mossambicus,* Oliveira and Almada [57] reported an increase in maternal aggressions during the development of the eggs and fry, becoming most intense when the young fish were capable of swimming freely outside the mouth of their mother. Data concerning sound production during this behaviour are unfortunately lacking for this species.

Female *O. niloticus* incubating eggs and embryos are unable to fight properly as a consequence of their mouth-breeding activity. During this period, female aggressiveness towards other individuals is reinforced by production of sounds. When the larvae are able to swim and feed by themselves, the female releases them while remaining able to take them back in the mouth in times of danger. When an intruder approaches, the first reaction of the female is not to recover its young but to directly attack the dangerous individual without any prior warning, threat or sound emission. This result also matches what is observed in *O. mossambicus,* where attack will prevail over control during this early phase in the development of the larvae [57]. In *A. centrarchus,* where the couple continues to emit sounds during the stage of free swimming larvae, the production of sounds by the parents does not seem to affect the behaviour of the young; the young themselves emit no sounds [10].

## Conclusion

This thorough approach to studying the acoustic behaviour of a cichlid species (*O. niloticus*) gave significant insight into their biology. Sounds may support some visual behaviours but are not systematically associated with any given behaviour. They are made by males and females in the context of territorial defence but do not seem to be related to courtship and mating. During agonistic interactions, sounds appear to be used to postpone or to lower aggressiveness: a calling *O. niloticus* does not simultaneously bite. In females, sounds may be made to defend the eggs but not the larvae. Comparisons with *O. mossambicus* highlight the fact that sounds in these two sister species can differ in usage, signature and function. This finding suggests that sounds may help in differentiating species and promoting speciation. It also makes clear that the functions of the sounds cannot be generalized to the entire taxa.
